# The gut microbiome and HLA-B27-associated anterior uveitis: a case-control study

**DOI:** 10.1186/s12974-024-03109-4

**Published:** 2024-05-07

**Authors:** Sophia C. Morandi, Elio L. Herzog, Marion Munk, Marco Kreuzer, Carlo R. Largiadèr, Sebastian Wolf, Martin Zinkernagel, Denise C. Zysset-Burri

**Affiliations:** 1 Department of Ophthalmology, Inselspital, Bern University Hospital, University of Bern, Bern, Switzerland; 2 Department for BioMedical Research, University of Bern, Bern, Switzerland; 3 Graduate School for Cellular and Biomedical Sciences, University of Bern, Bern, Switzerland; 4 Interfaculty Bioinformatics Unit, University of Bern, Bern, Switzerland; 5 Department of Clinical Chemistry, Bern University Hospital, Inselspital, University of Bern, Bern, Switzerland

**Keywords:** Gut microbiome, Anterior uveitis, HLA-B27, Whole metagenome shotgun sequencing, *Eubacterium ramulus*, *Phocaeicola vulgatus*, *Bacteroides caccae*, Lipid IVA biosynthesis

## Abstract

**Background:**

The human gut microbiome (GM) is involved in inflammation and immune response regulation. Dysbiosis, an imbalance in this ecosystem, facilitates pathogenic invasion, disrupts immune equilibrium, and potentially triggers diseases including various human leucocyte antigen (HLA)-B27-associated autoinflammatory and autoimmune diseases such as inflammatory bowel disease (IBD) and spondyloarthropathy (SpA). This study assesses compositional and functional alterations of the GM in patients with HLA-B27-associated non-infectious anterior uveitis (AU) compared to healthy controls.

**Methods:**

The gut metagenomes of 20 patients with HLA-B27-associated non-infectious AU, 21 age- and sex-matched HLA-B27-negative controls, and 6 HLA-B27-positive healthy controls without a history of AU were sequenced using the Illumina NovaSeq 6000 platform for whole metagenome shotgun sequencing. To identify taxonomic and functional features with significantly different relative abundances between groups and to identify associations with clinical metadata, the multivariate association by linear models (MaAsLin) R package was applied.

**Results:**

Significantly higher levels of the *Eubacterium ramulus* species were found in HLA-B27-negative controls (*p* = 0.0085, Mann-Whitney U-test). No significant differences in microbial composition were observed at all other taxonomic levels. Functionally, the lipid IV_A_ biosynthesis pathway was upregulated in patients (*p* < 0.0001, Mann-Whitney U-test). A subgroup analysis comparing patients with an active non-infectious AU to their age- and sex-matched HLA-B27-negative controls, showed an increase of the species *Phocaeicola vulgatus* in active AU (*p* = 0.0530, Mann-Whitney U-test). An additional analysis comparing AU patients to age- and sex-matched HLA-B27-positive controls, showed an increase of the species *Bacteroides caccae* in controls (*p* = 0.0022, Mann-Whitney U-test).

**Conclusion:**

In our cohort, non-infectious AU development is associated with compositional and functional alterations of the GM. Further research is needed to assess the causality of these associations, offering potentially novel therapeutic strategies.

**Supplementary Information:**

The online version contains supplementary material available at 10.1186/s12974-024-03109-4.

## Background

Uveitis is an intraocular inflammation that is estimated to account for 10–15% of cases of blindness in the developed world [1]. Etiologically, uveitis can be classified into infectious and non-infectious types, the latter accounting for up to 90% of all uveitis cases in highly industrialized countries [2, 3]. The main drivers of non-infectious uveitis seem to be a loss of control mechanism or regulation of both, the innate immune system, leading to autoinflammation, and the adaptive immune system, leading to autoimmunity [4, 5]. Anterior uveitis (AU) is the most common type of non-infectious uveitis and affects the anterior part of the eye including the iris, the ciliary body, and the anterior chamber [2, 6].

Non-infectious AU is a polygenic disease associated with various human leukocyte antigen (HLA) alleles, with HLA-B27 being the most common [7]. HLA-B27 is connected with other autoinflammatory and autoimmune diseases such as inflammatory bowel disease (IBD) and spondyloarthropathy (SpA) in 25–78% of cases as well [8]. The HLA system is the human form of a major histocompatibility complex (MHC) and plays a key role in the differentiation between exogenous and endogenous structures by the immune system via MHC-restricted antigen recognition [9]. It is critical for self-tolerance, ensuring that the immune system avoids targeting the body’s own tissues. It is assumed that HLA-B27 may impact the development of autoimmune disorders by modulating the immune response to specific microbial antigens and is associated with both, intestinal tolerance as well as the loss of ocular immune privilege occurring in acute AU, but it is not completely understood how [10].

Recent studies suggest a significant role of gut microbiome (GM) in the pathogenesis of HLA-B27-associated non-infectious AU [11–13]. The human GM is a complex and non-negligible ecosystem of microorganisms–- including bacteria, viruses, and fungi–- as well as their combined genetic material [14]. Microbes in the human body make up to 100 trillion cells (a number which is 10 times higher than the number of human cells) and most of them reside in the gut [15]. As such, the GM provides many important physiological functions, including not only the promotion of digestion and absorption of nutrients or the synthesis of vitamins and amino acids but also inflammation and immune homeostasis [16]. Aberrant microbial composition or function of the GM, also known as dysbiosis, can favor the invasion and growth of pathogenic species, promote systemic inflammation, disrupt immune homeostasis, and thus potentially induce diseases [16].

In 2014, a study conducted on Lewis-strain rats that are transgenic for HLA-B27 and the human β2-microglobulin showed the influence of HLA-B27 on the GM [17]. The human β2-microglobulin is a component of the class I MHC involved in the presentation of peptide antigens to the immune system. Specific MHC haplotypes have been identified to contribute to shaping an individual’s unique microbial composition, potentially influencing their susceptibility to intestinal infections [18]. Furthermore, associations have been discovered between the GM and HLA-B27-associated diseases such as IBD and SpA [13, 19]. Further, research investigating a so-called “gut-eye axis” has revealed an increasing number of interconnections between the GM and several other eye diseases such as age-related macular degeneration, glaucoma, and retinal artery occlusion [20–23].

Previous research highlights the complexity of the mechanisms and systemic interconnections on the “gut-eye axis” that seem to be at play. Approaches that attempt to explain how HLA-B27 affects GM and in turn predisposes to AU name a variety of mechanisms such as HLA-B27-associated dysbiosis, increased gut permeability, and molecular mimicry of HLA-B27 restricted microbial antigens leading to an aberrant immune response [10, 24]. However, to untangle the complex systemic interplay leading to HLA-B27-associated AU and the potential implications of the GM, more research is needed including studies that triangulate data on AU, the GM, and HLA-B27.

This study presents the characterization of the GM in patients with HLA-B27-associated non-infectious AU compared to HLA-B27-negative and HLA-B27-positive healthy controls. The findings are expected to provide an improved basis for a better understanding of the complex causes of AU.

## Methods

### Study aim, design, and setting

To assess if the GM is associated with HLA-B27-associated non-infectious AU, a cross-sectional case-control study was performed. It is based on the collection of stool samples from 47 study participants.

Twenty individuals were HLA-B27-positive and had a history of clinically confirmed acute AU. Their GM was compared to the GM of 21 age- and sex-matched HLA-B27-negative controls with no history of AU or any other type of uveitis (Table 1). For the analysis, no differentiation between different types of HLA-B27-associated non-infectious AU was made. Within the group of HLA-B27-associated AU patients, 8 out of 20 were diagnosed with a form of SpA and 7 out of 20 were under treatment with systemic steroids and/or synthetic or biologic disease-modifying anti-rheumatic drugs (DMARDs). For the extended data on the AU patient population, including disease status, HLA-B27-associated systemic diseases, therapy, anterior chamber cell grading schema according to the SUN grading system, and the year of the initial AU diagnosis and recurrency see supplementary data (Table 1s).

A subgroup analysis was conducted comparing seven patients (among the total of 20 patients) with an active AU during the time of sampling to seven age- and sex-matched healthy HLA-B27-negative controls (Table 2).

In an additional analysis, six healthy HLA-B27-positive controls were compared to six age- and sex-matched patients (among the total of 20 patients; see Table 3).

**Table 1:** Characteristics of the study group – HLA-B27-associated AU vs. HLA-B27-negative CTRL

Feature	Patients (*n* = 20)	Controls (*n* = 21)	*p*-value
Male/Female (n)	11/10	11/11	> 0.9999^a^
Mean Age (years)	M = 44.5, SD = 16.3	M = 42.1, SD = 14.9	0.6268^b^
BMI (kg/m2)	M = 25.4, SD = 5.2	M = 22.4, SD = 2.8	0.0305^b^

Data show mean and SD (standard deviation); BMI (body mass index); CTRL (control group)

^a^Fisher’s exact test. ^b^Welch’s t-test

**Table 2:** Characteristics of the study subgroup – HLA-B27-associated active AU vs. HLA-B27-negative CTRL

Feature	Patients (*n* = 7)	Controls (*n* = 7)	*p*-value
Male/Female (n)	3/4	3 /4	> 0.9999^a^
Age (years)	M = 47.4, SD = 7.1	M = 45.9, SD = 16.0	0.8722^b^
BMI (kg/m2)	M = 26.7, SD = 3.4	M = 22.0, SD = 2.1	0.0155^b^

Data show mean and SD (standard deviation); BMI (body mass index); CTRL (control group) ^a^Fisher’s exact test. ^b^Welch’s t-test

**Table 3:** Characteristics of the additional study group – HLA-B27-associated AU vs. HLA-B27-positive CTRL

Feature	Patients (*n* = 6)	Controls (*n* = 6)	*p*-value
**Male/Female (n)**	2/4	2/4	> 0.9999^a^
**Age (years)**	M = 51.2, SD = 16.7	M = 50.3, SD = 15.6	0.9366^b^
**BMI (kg/m2)**	M = 24.7, SD = 3.7	M = 21.8, SD = 3.4	0.2217^b^

Data show mean and SD (standard deviation); BMI (body mass index); CTRL (control group) ^a^Fisher’s exact test. ^b^Welch’s t-test

All 47 participants were aged 18 years or older and were able to give informed consent. They were recruited from either the ward or the retina outpatient clinic in the Department of Ophthalmology at the University Hospital Bern (Inselspital), located in Switzerland from 2016 until 2021. The study was approved by the local ethics committee (NCT02438111) and adheres to the Declaration of Helsinki.

Exclusion criteria for both groups were chronic IBD, rheumatoid arthritis, lupus erythematosus, smoking, diabetes, treated hyperlipidemia, a history of use of systemic antibiotics within the last 3 months and opacities of the ocular media that occlude detailed observations of the retina.

### HLA-B27 typing

The certified HLA-Ready Gene B27-Kit (cat. no. 002 058 032; inno-train Diagnostik GmbH, Kronberg, Germany) based on the Single Specific Primer-Polymerase Chain Reaction (SSP-PCR) technology, was used for screening patients for HLA-B27 alleles (HLA-B27:02, *27:04, *27:05, *27:14, *27:06, *27:07, *27:09).

### Metagenomic DNA isolation, sequencing, and data quality control

Stool samples were delivered refrigerated to the research facility within 16 h after defecation and were promptly frozen at -20 °C. Metagenomic DNA isolation and sequencing were performed following the protocol outlined in Zysset-Burri et al. [22]. This included the use of the TruSeq DNA PCR-Free Library Preparation kit for library preparation and the Illumina NovaSeq 6000 platform of the University of Bern, Switzerland, for whole metagenome shotgun sequencing.

The reads were filtered using KneadData v0.10.0 (https://huttenhower.sph.harvard.edu/kneaddata/), which includes trimming low-quality reads, and removing host and rRNA sequences.

### Microbial and functional profiling

The taxonomic and functional profiles of the microbial communities were determined as described in Zysset-Burri et al. [22]. This included the use of Metagenomic Phylogenetic Analysis v.2.6.0 and v.3.14 (MetaPhlAn2 and MetaPhlAn3) with marker database v.20 [25] and the Unified Metabolic Analysis Network (HUMAnN3 v.3.0.1.0) [26]. MetaPhlAn resulted in a relative abundance of microbial species (taxonomic profiling) and HUMAnN2 in relative abundance of functional pathways and gene families (functional profiling).

### Statistical analysis

Demographics were compared among groups applying either Fisher’s exact test (for sex) or Welch’s t-test (for age and BMI) in GraphPad Prism (Version 8.0.1) (GraphPad Software Inc.). P-values < 0.05 were considered statistically significant.

R software (version 4.2.2) and GraphPad Prism were used to compare the microbial and pathway abundances between HLA-B27-associated non-infectious AU patients and HLA-B27-negative controls. We employed the R package vegan v.2.6.4 for Shannon’s diversity analysis and the package ade4 v.1.7.22 for principal component analysis (PCA) [27]. For separation assessment, permutation multivariate analysis of variance (PERMANOVA) using the R package vegan was performed with 10’000 permutations, resulting in a calculated p-value [28].

To identify associations between microbial and pathway abundances clinical metadata (sex, age, and BMI), the multivariate analysis by linear models (MaAsLin2) R package [29] was applied with adjustments of the default settings to “no log transformation”, q < 0,02 and *N* ≠ 0 in over 50%, as well as the Mann-Whitney U-test (for groups and sex) and the linear regression model (for age and BMI) in GraphPad Prism.

Enterotyping was performed according to the tutorial and R code of Arumugam et al. [30].

## Results

### Taxonomic characterization of the GM of HLA-associated non-infectious AU patients and HLA-B27-negative controls

In total, we generated 1.60 billion 151 bp paired-end reads with a mean of 34.71 million (SD = 10.78 million) reads per sample. Following trimming and filtering, we obtained a mean of 33.67 million (SD = 10.38 million) non-human high-quality reads per sample for further processing.

**Fig. 1:**
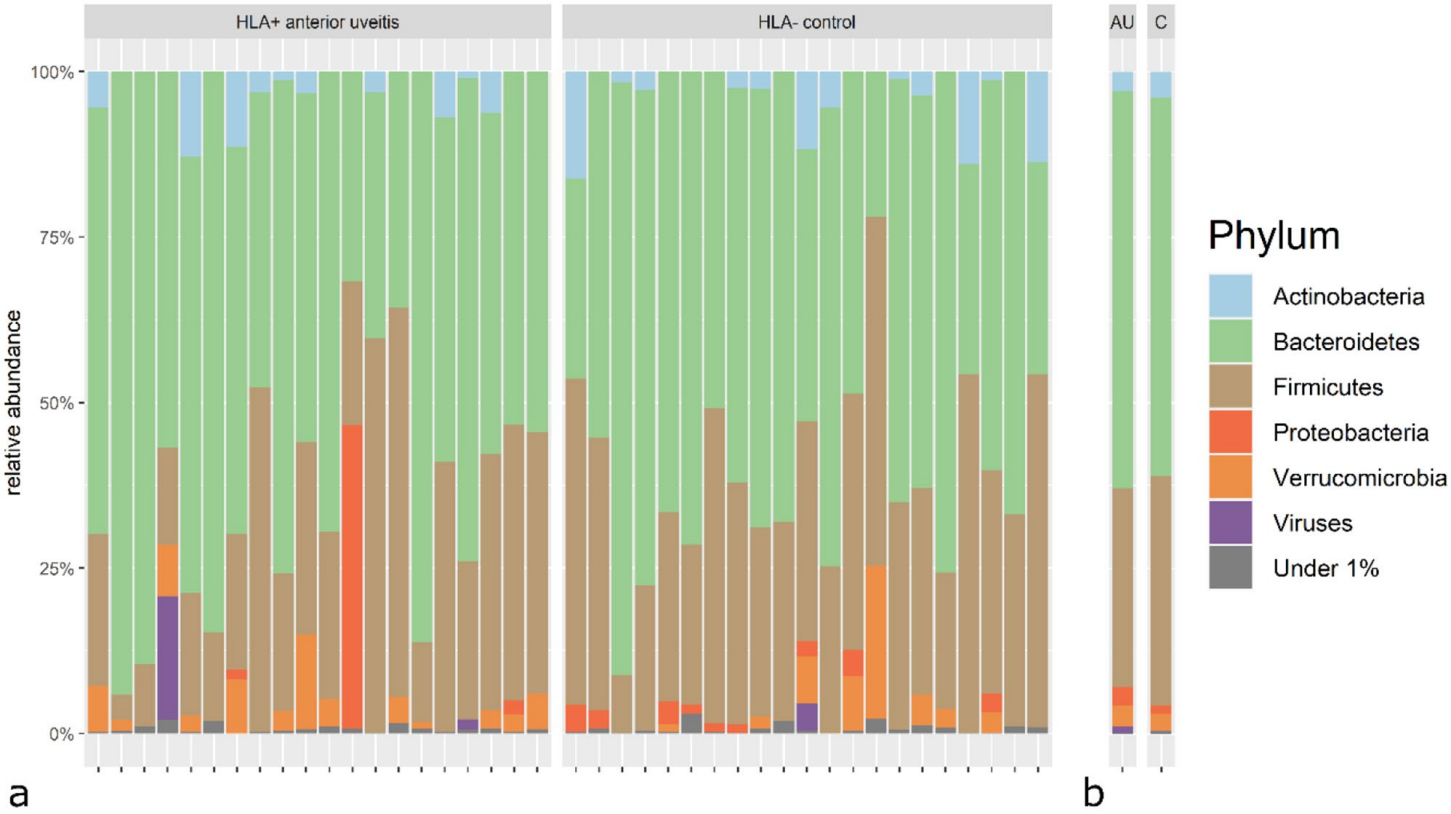
Taxonomic characterization of the GM composition of the study group. Relative abundances of all phyla in HLA-B27- associated non-infectious AU patients (*n* = 20) and HLA-B27-negative controls (*n* = 21) (**a**), averaged for study groups (**b**)

The majority of the microbial reads were from bacteria (M = 98.80%, SD = 4.25% in patients and M = 99.65%, SD = 0.90% in controls), primarily belonging to the phyla *Bacteroidetes* (M = 61.50%, SD = 17.67% in patients and M = 57.25%, SD = 17.05% in controls) and *Firmicutes* (M = 28.38%, SD = 15.80% in patients and M = 34.56%, SD = 11.62% in controls), followed by the phyla *Verrucomicrobia* (M = 3.21%, SD = 3.66% in patients and M = 2.55%, SD = 5.19% in controls), *Actinobacteria* (M = 2.95%, SD = 3.71% in patients and M = 3.85%, SD = 5.08% in controls), *Proteobacteria* (M = 2.74%, SD = 9.90% in patients and M = 1.30%, SD = 1.34% in controls) and viruses (M = 1.12%, SD = 4.03% in patients and M = 0.32%, SD = 0.89% in controls) (Fig. 1).

The Most abundant classes in the study group were *Bacteroidia* and *Clostridia*, the most abundant genera were *Bacteroides, Alistipes*, *Prevotella,* and *Faecalibacterium*; finally, the most abundant species were *Prevotella copri*, *Alistipes putredini*s, *Bacteroides uniformis, Faecalibacteria prausnitzi,i* and *Phocaeicola vulgatus* (Table 2s, supplementary data).

### Differences in composition and function in HLA-B27-associated non-infectious AU compared to HLA-B27-negative controls

No significant differences in α-diversity were found between patients and HLA-B27-negative controls with 341 species overall, of which 286 species were found in patients (23 species with a RA > 1%) and 273 species in controls (22 species with a RA > 1%).

The Shannon diversity index was not significantly different between the two groups, with a mean index of 2.44 in patients and 2.53 in controls (*p* = 0.58, Welch’s two-sample t-test).

PCA using the health status as grouping variable did not result in differences between patients and controls based on differences in the relative abundance of microbial species (*p* = 0.76, R² = 0.02, PERMANOVA analysis with nb of permutations = 10,000) nor in relative abundance of pathways (*p* = 0.27, R² = 0.03, PERMANOVA analysis with nb of permutations = 10,000). Subgroup analysis comparing only patients with an active non-infectious AU at the time of sampling with their age- and sex-matched controls showed the same results for species (*p* = 0.65, R² = 0.06, PERMANOVA analysis with nb of permutations = 10,000) and pathways (*p* = 0.37, R² = 0.08, PERMANOVA analysis with nb of permutations = 10,000).

**Fig. 2:**
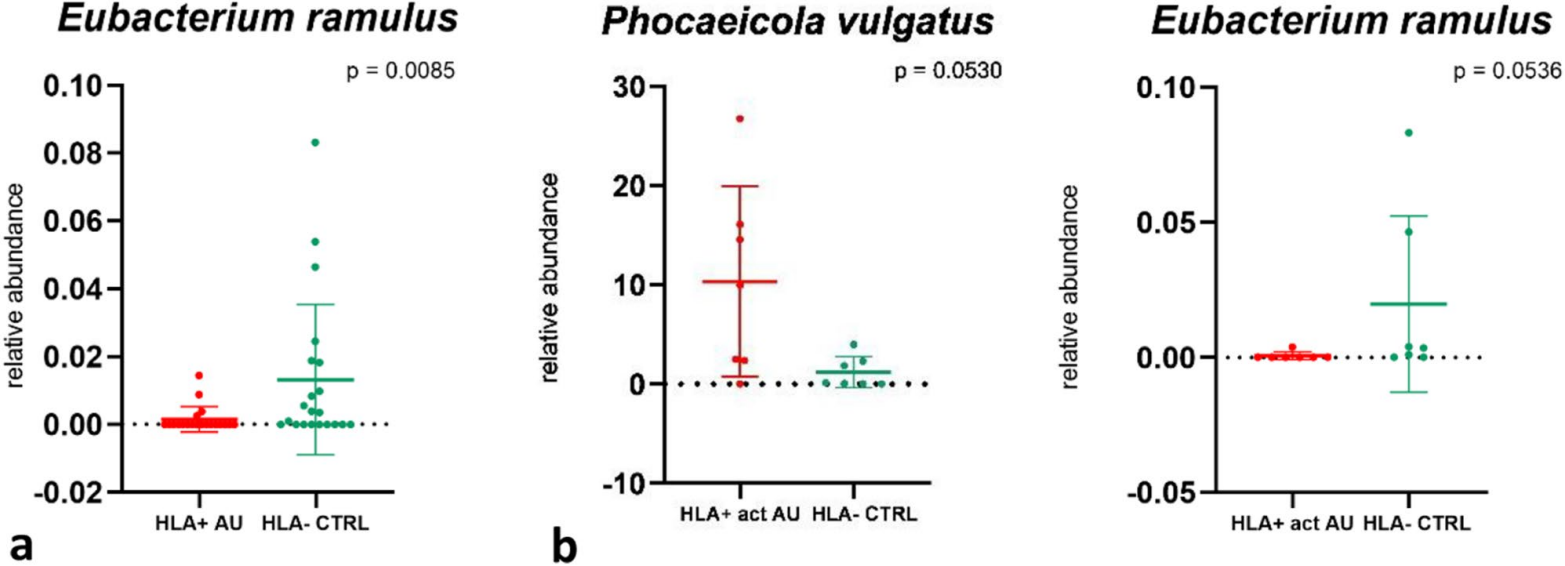
Differences in microbial composition between patients and controls at species level. Comparisons between patients with HLA-B27-associated non-infectious AU and HLA-B27-negative controls (**a**). Subgroup analysis comparing patients with an HLA-B27-associated active AU at the time of sampling to controls (**b**). Dot plots show the relative abundance, mean, and SD of the species that were different between patients and controls. In the group analysis, *Eubacterium ramulus* was significantly increased in controls, while in the subgroup analysis *Phocaeicola vulgatus* was increased in patients with active AU

No significant differences in microbial composition between patients and controls were observed at all taxonomic levels except at species level. The GM of controls was significantly increased in the species *Eubacterium ramulus* (*p* = 0.0085, Mann-Whitney U-test) (Fig. 2a). The subgroup analysis showed an increase of *Eubacterium ramulus* (*p* = 0.0536, Mann-Whitney U-test) in controls and an increase of *Phocaeicola vulgatus* (formerly called *Bacteroides vulgatus*) in the active non-infectious AU group (*p* = 0.0503, Mann-Whitney U-test) compared to controls (Fig. 2b).

**Fig. 3:**
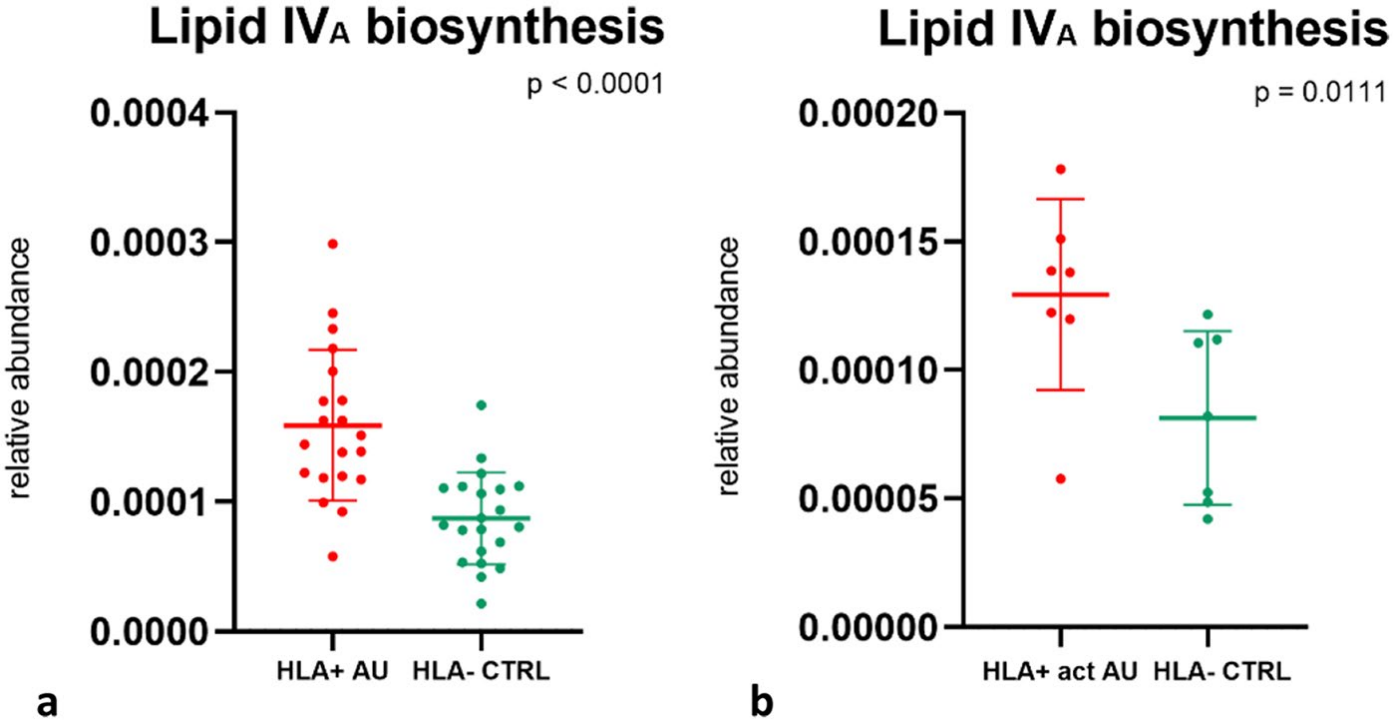
Differences in pathway expression between patients and controls. Comparisons between patients with HLA-B27-associated non-infectious AU and HLA-B27-negative controls (**a**). Subgroup analysis comparing patients with an active AU at the time of sampling to HLA-B27-negative controls (**b**). Dot plots show the relative abundance, mean and SD of the pathways significantly different between patients and controls. Lipid IV_A_ biosynthesis was upregulated in patients in both, group and subgroup analysis

Functionally, the lipid IV_A_ biosynthesis pathway, a precursor of lipid A in the biosynthesis of lipopolysaccharide (LPS) [31], was upregulated in patients compared to controls in the group analysis (*p* < 0.0001, Mann-Whitney U-test) (Fig. 3a) as well as in the subgroup analysis (*p* < 0.0111, Mann-Whitney U-test) (Fig. 3b).

No associations were found between species and pathway abundances, and age, sex, and BMI.

### Additional analysis: differences in composition in HLA-B27-associated non-infectious AU compared to HLA-B27-positive controls

**Fig. 4:**
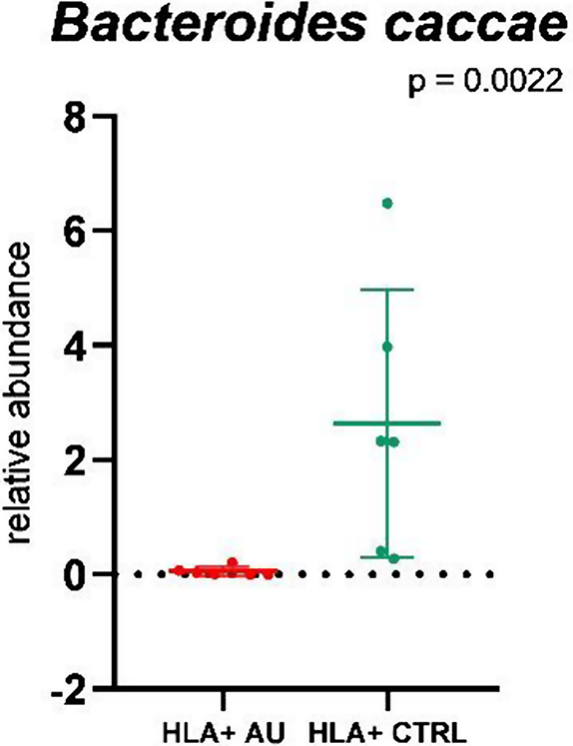
Differences in microbial composition between patients and HLA-B27-positive controls at species level. Comparisons between patients with HLA-B27-associated non-infectious AU and HLA-B27-positive controls. Dot plots show the relative abundance, mean, and SD of the species that were different between patients and controls. *Bacteroides caccae* was significantly increased in controls

The analysis of the GM of HLA-B27-positive AU patients and HLA-B27-positive controls showed an increase of *Bacteroides caccae* in controls (*p* = 0.0022, Mann-Whitney U-test) (Fig. 4).

No associations were found between species abundances and age, sex, and BMI.

### Enterotypes classification of the HLA-B27-positive non-infectious AU and HLA-B27-negative controls

Previous studies have indicated that the human GM can be classified into distinct enterotypes based on their specific microbial compositions [30]. In line with these findings, we identified four enterotypes and three driving genera (Fig. 2). Results are based on the application of the Jensen-Shannon distance for the relative abundance of the genera and employed partitioning around medoids (PAM) to cluster the samples. The optimal number of clusters was calculated using the Calinski-Harabasz (CH) index (Fig. 2a). The results of the between-class-analysis (BCA) visualized the taxonomic factors driving the clusters and showed the four clusters (*p* < 0.001, R² = 0.499, PERMANOVA analysis with nb of permutations = 10,000). Clusters 1, 3, and 4 were mainly driven by the following individual genera: enterotype 1 by *Bacteroides*, enterotype 3 by *Prevotella,* and enterotype 4 by *Alistipes*, whereas cluster 2 was a mixed cluster driven by all four of the genera (Fig. 2b and c).

**Fig. 5:**
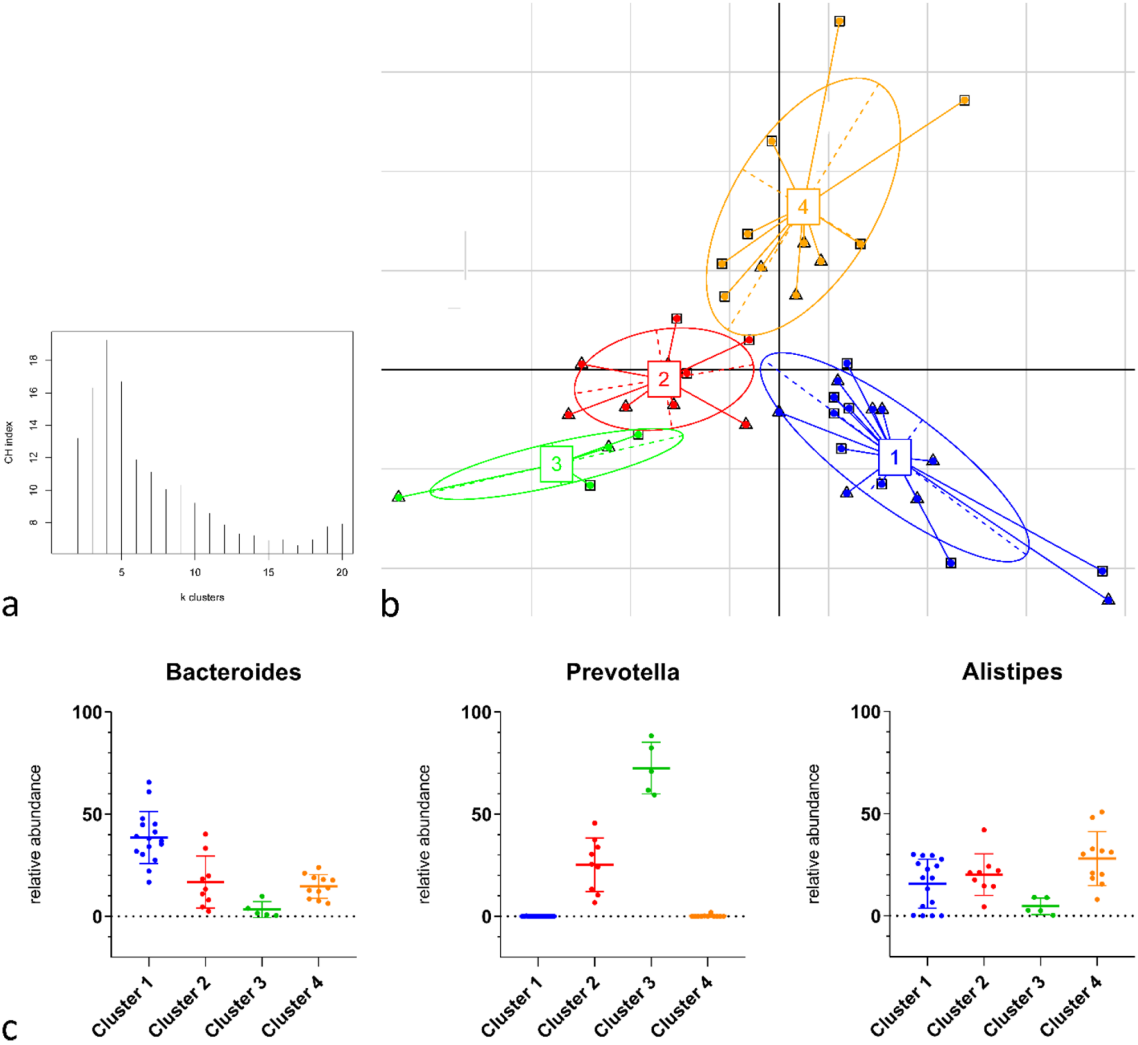
Enterotype categorization of the study groups at genera level. BCA showed four enterotype clusters in the study groups. Patients (*n* = 20) are marked by triangles (∆) and controls (*n* = 21) by squares (□) (**a**). Calinski-Harabasz (CH) index was used to calculate the optimal number of clusters (**b**). Dot plots show the relative abundance, mean, and SD of the three genera identified to characterize the four enterotypes (**c**). The colors for the four enterotypes are blue for 1, red for 2, green for 3, and orange for 4

However, we identified no significant association between the enterotypes and HLA-B27-associated AU. The samples of patients and controls were evenly distributed among the four enterotypes (See Fig. 5).

## Discussion

This study revealed no significant difference in α-diversity and Shannon’s diversity index among HLA-B27-associated non-infectious AU patients compared to healthy HLA-B27-negative controls. PCA did not show any separation between groups and subgroups. There were no significant differences in microbial composition between patients and controls at all taxonomic levels except at species level. However, we identified one specific species and one pathway with a significantly different relative abundance in patients and controls.

The gram-positive species *Eubacterium ramulus* was increased in HLA-B27-negative controls compared to patients. It is a widespread and beneficial gut microbe able to metabolize various dietary flavonoids [32]. Flavonoids are polyphenolic plant compounds, which are believed to have therapeutic effects due to their anti-oxidative, anti-inflammatory, and anti-viral properties [33]. A 12-week pilot study tested probiotics as supplementary therapy alongside mesalazine in patients with ulcerative colitis (a form of IBD) and found the symptom alleviation to be significantly greater in the probiotics group compared to the mesalazine-only placebo group. Amongst the 16 species found exclusively in the probiotics group was *Eubacterium ramulus* [34]. Thus, this species might have a protective effect against autoinflammatory and autoimmune disease.

Furthermore, the subgroup analysis, comparing the GM of active AU patients to the GM of inactive AU patients, showed an increased amount of *Phocaeicola vulgatus* within patients with active AU. *Phocaeicola vulgatus* is gram-negative and one of the most common species of the *Bacteroide*s genus, which constitutes 30% of the colonic bacteria [35]. AU is often a recurrent disease with active and inactive phases. *Phocaeicola vulgatus* may play a role in triggering and/or sustaining disease activity. In the previously mentioned study conducted on Lewis strain HLA-B27/human β2-microglobulin transgenic rats developing severe arthritis but no gut inflammation, species-specific differences in the GM included an increase in *Phocaeicola vulgatus* abundance [17]. Earlier studies in (non-Lewis) HLA-B27 transgenic rats showed that a germ-free environment prevented both, gut and joint inflammation which otherwise occur spontaneously [36], and that a mono-colonization with *Phocaeicola vulgatus* sufficed to induce colitis [37]. The studies suggest that the interaction between specific species such as *Phocaeicola vulgatus* and HLA-B27 increases the vulnerability to autoinflammatory and autoimmune disease. A multi-omics study found that a subset of patients with ulcerative colitis had an abnormally high abundance of *Phocaeicola vulgatus* proteases (mostly serine proteases), which in turn correlated positively with the disease severity and activity. Moreover, stool transplants from patients with high levels of these proteases induced colitis in germ-free mice. Inversely, inhibition of these proteases improved intestinal barrier dysfunction and prevented colitis in these mice as well as in *Phocaeicola vulgatus* monocolonized, IL-10 deficient mice [38]. This multi-omics study show that it might not be the presence of *Phocaeicola vulgatus* itself but the increase of its proteases that might trigger and/or sustain AU activity and highlights the interest of multi-omic approaches in further studies linking AU with the GM.

Secondly, this study revealed an upregulation of the lipid IV_A_ biosynthesis pathway in patients compared to HLA-B27-negative healthy controls, in both, group and subgroup analysis. As mentioned previously, lipid IV_A_ is a tetra-acylated precursor of lipid A in the biosynthesis of LPS. LPS is a major outer-membrane component of gram-negative bacteria and constitutes an important activator of innate immune responses [31]. With the establishment of the endotoxin-induced uveitis model in rodents in 1980 it has been shown that LPS facilitates inflammation of the uvea [39]. The mechanism involves LPS activating Toll-like receptors, by acting as a pathogen-associated molecular patterns [40]. For instance, LPS binds to the Toll-like receptor 4 (TLR4)-MD2 receptor complex, activating pro-inflammatory signaling pathways. Therefore, researchers have suggested targeting this pathway as a potential strategy to regulate inflammation [41]. Nevertheless, the analysis of pathways detected by gene sequencing is not enough to confirm their relevance, because the mere presence of pathway encoding DNA does not automatically equate to active protein synthesis and functionality.

The BMI was significantly increased in patients compared to HLA-B27 negative controls in both, the group and the subgroup. Recent studies suggest that obesity may contribute to AU [42]. *Eubacterium ramulus* and its metabolites are believed to contribute to the reduction of obesity, whereas *Phocaeicola vulgatus* is controversially discussed in this regard [43–46]. However, we did not find any significant correlation between the GM and metadata including BMI, suggesting that the differences we show in our study are attributed to the disease and not to the difference in BMI.

The additional analysis comparing HLA-B27-associated non-infectious AU patients to healthy HLA-B27-positive controls showed an increased amount of *Bacteroides caccae* within the HLA-B27-positive controls. *Bacteroides caccae* is a common gut microbe and has been identified as the most prevalent strain shared between mothers and children in a Scandinavian cohort [47]. In IBD, characterized by an aberrant immune response to endogenous gut microbes, *Bacteroides caccae* has been identified to trigger strong serological responses implicating the IBD-associated monoclonal antibody pANCA (perinuclear anti-neutrophilic antibody) and *Bacteroides caccae*’s TonB-linked outer membrane protein OmpW [48–51].

*Bacteroides caccae* may therefore play a protective role in the development of AU in HLA-B27-positive individuals on one hand and may increase the risk of developing a form of IBD on the other hand.

This hypothesis is supported by the observation that although both AU and IBD are associated with HLA-B27, the eye is only the third most commonly affected extraintestinal tissue in IBD patients with 2–5%, whereas musculoskeletal and cutaneous manifestations occur in 40% and 15% of patients, respectively. Moreover, amongst the ocular manifestations, uveitis occurs independently of intestinal-IBD activity, whereas episcleritis and scleritis correlate with intestinal disease activity [52].

Patients and controls were evenly distributed amongst the four enterotypes identified in this study, indicating no association between enterotypes and HLA-B27-associated AU. We did not collect diet data and did not analyze which other demographic variables might explain the four enterotypes as this was out of the scope of this study.

Overall, our results show that the GM of HLA-B27-positive non-infectious AU patients versus healthy HLA-B27-positive and -negative controls are altered in specific species, which in turn may trigger or sustain the activity of the disease.

### Further directions

Previous studies emphasize the importance of well-controlled investigations and the involvement of various factors alongside the GM in the disease development of HLA-B27-associated uveitis [24]. In this study, we included a subgroup analysis comparing the GM of patients with an active AU to the GM of controls. This subgroup analysis suggests specific changes in the GM when the disease is in remission compared to when it is in its inactive phase, which has been shown to be the case in SpA [53]. Longitudinal studies of the GM in patients with AU may reveal a change in the GM in various phases of the disease. This may allow a better understanding of how the GM, AU, and disease activity are connected and whether a change in the GM may trigger, perpetuate, reactivate, or prevent AU.

Furthermore, while patients with IBD were excluded, other diseases associated with HLA-B27 were not. Studies have demonstrated that HLA-B27-associated disease such as Ankylosing spondylitis, a form of SpA, is also associated with changes in the GM. It seems important to compare HLA-B27-associated AU without associated systemic disease to HLA-B27-associated AU in patients with forms of IBD or SpA to elucidate their potential role as confounding factors in future studies [54, 55]. In this context, it is important to note that several patients with SpA suffer from an underlying IBD [56].

Moreover, although patients with a history of antibiotic therapy within the last three months were excluded from the study, patients with systemic steroids and/or synthetic or biologic disease-modifying anti-rheumatic drugs (DMARDs) were not. Further research on AU should consider the potential impact of systemic immunomodulatory medication on the GM, as emerging studies suggest that these medications possess gut microbiota-modifying properties. For instance, steroids have been associated with changes in the GM, and therapy with the tumor necrosis factor-inhibitor (TNFi), a biologic DMARD, was correlated with restoration of the perturbed GM in ankylosing spondylitis [57, 58]. Secondly, the gut microbiome may play a role in immunotherapy efficacy: For instance, in cancer immunotherapy, there is growing recognition that the gut microbiome influences antitumor therapy response [59, 60]. Despite these potentially confounding factors, our study revealed a difference between patients and controls. However, the inability to exclude the influence of HLA-B27-associated diseases as well as of medication on the GM in AU is a limitation of our study.

Limitations are also given by the small sample size of this study, as well as the restricted geographical location of the study subjects to Switzerland. Further studies are needed to confirm the findings of this study based on a larger database and in different geographical contexts. Finally, yet importantly, future research endeavors should strive to elucidate, which specific GM alterations are attributed to the HLA-B27 genotype and which to AU activity. Current evidence suggests that changes in the GM in other HLA-B27-associated autoimmune diseases are partly due to disease activity and partly to the effects of HLA-B27 [61, 62]. Disparities in the GM between HLA-B27-positive and HLA-B27-negative autoimmune disease-affected patients have also been noted in previous research [63, 64].

## Conclusion

The results of this study suggest that HLA-B27-associated non-infectious AU development is influenced by compositional and functional alterations of the GM. An increase in specific gram-negative bacteria and LPS might play a role as a trigger for inflammation and aberrant immune response in the eye. Further research is needed to prove the causality of these connections, offering potentially novel microbiome-altering therapeutic strategies for AU.

### Electronic supplementary material

Below is the link to the electronic supplementary material.

Supplementary Material 1

## Data Availability

The datasets evaluated in this study are available in the European Nucleotide Archive under accession number PRJEB55787.

## Abbreviations

AU: Anterior uveitis
BMI: Body mass index
GM: Gut microbiome
HLA: Human leukocyte antigen
IBD: Inflammatory bowel disease
LPS: Lipopolysaccharide
MHC: Major histocompatibility complex
PERMANOVA: Permutation multivariate analysis of variance
RA: Relative abundance
SpA: Spondyloarthropathy
