# Supplementary material for: The gut microbiome and HLA-B27-associated anterior uveitis: a case-control study
Source: J Neuroinflammation. 2024 May 7;21:120. doi: 10.1186/s12974-024-03109-4 (PMC11077820; doi:10.1186/s12974-024-03109-4)
Supplement: Supplementary file 1 — Supplementary Material 1 [file 12974_2024_3109_MOESM1_ESM.docx]

**Supplementary Data**

**Table 1s. Extended data on the AU patient population**

| **Group and subgroups** | **Disease status** | **AC cells** | **Single episode / recurrent** | **HLA-B27-associated systemic disease** | **Therapy** |
| --- | --- | --- | --- | --- | --- |
| AU, active AU | Active | 2+ | single episode | Ankylosing spondylitis | - |
| AU | Not active | - | recurrent since 2015 | - | - |
| AU, active AU | Active | 1+ | recurrent since 2018 | - | - |
| AU | Not active | - | recurrent since 2015 | - | - |
| AU | Not active | - | recurrent since 2016 | - | - |
| AU | Not active | - | recurrent since 2020 | - | - |
| AU, active AU | Active | 2+ | recurrent since 2018 | Ankylosing spondylitis | - |
| AU | Not active | - | recurrent since 2006 | - | - |
| AU | Not active | - | recurrent since 2019 | - | Prednison |
| AU | Not active | - | single episode | Juvenile idiopathic arthritis | Methotrexat, Humira |
| AU | Not active | - | recurrent since 2008 | - | Humira |
| AU | Not active | - | recurrent since 2017 | Spondyloarthropathy | Simponi |
| AU | Not active | - | recurrent since 2019 | Spondyloarthropathy | - |
| AU | Not active | - | recurrent since 2012 | Ankylosing spondylitis | Humira |
| *AU, active AU, additional analysis* | *Active* | *0.5+* | *recurrent since 1994* | *-* | *Methotrexat, Prednison, Humira* |
| *AU, additional analysis* | *Not active* | *-* | *recurrent since 1991* | *-* | - |
| *AU, active AU, additional analysis* | *Active* | *0.5+* | *recurrent since 2008* | *Spondyloarthropathy* | - |
| *AU, active AU, additional analysis* | *Active* | *0.5+* | *recurrent since 2010* | *Spondyloarthropathy* | - |
| *AU, active AU, additional analysis* | *Active* | *1+* | *recurrent since 2018* | *-* | *Hyrimoz* |
| *AU, additional analysis* | *Not active* | *-* | *recurrent since 2016* | *-* | - |
| AC cells: AC cell grading schema according to SUN grading system. AC (anterior chamber); SUN (Standardization of Uveitis Nomenclature); i.d. (initial diagnosis) | | | | | |

**Table 2s: Microbial composition at class genus and species level**

| **Class** | **Patients (M, SD)** | **Controls (M, SD)** |
| --- | --- | --- |
| Bacteroidia | 61.50, 17.67 | 57.25, 17.05 |
| Clostridia | 24.08, 15.44 | 28.67, 10.70 |
| **Genus** | **Patients (M, SD)** | **Controls (M, SD)** |
| Bacteroides | 26.24, 16.83 | 20.11, 15.49 |
| Alistipes | 17.71, 11.22% | 19.62, 14.34 |
| Prevotella | 15.15, 24.06 | 9.26, 7.81 |
| Faecalibacterium | 13.81, 25.77 | 7.97, 4.51 |
| **Species** | **Patients (M, SD)** | **Controls (M, SD)** |
| Prevotella copri | 13.21, 23.30 | 13.47, 25.87 |
| Alistipes putredinis | 12.98, 11.30 | 10.71, 12.22 |
| Bacteroides uniformis | 8.48, 7.05 | 5.09, 4.74 |
| Faecalibacterium prausnitzii | 8.21, 6.41 | 7.97, 4.51 |
| Bacteroides vulgatus | 6.51, 7.02 | 7.34, 9.75 |

Table shows mean (M) RA [%] and standard deviation (SD) of RA [%]
